# Fogarty Catheter Placement for Subglottic H-Type Tracheoesophageal Fistula via a Supraglottic Airway: A Case Report

**DOI:** 10.1155/crpe/6785603

**Published:** 2025-11-18

**Authors:** Akihisa Kawamura, Machiko Furuta, Shugo Kasuya

**Affiliations:** ^1^Department of Anesthesiology, University of Tsukuba Hospital, 2-1-1 Amakubo, Tsukuba-shi, Ibaraki 305-8576, Japan; ^2^Department of Critical Care and Anesthesia, National Center for Child Health and Development, 2-10-1 Okura, Setagaya-ku, Tokyo 157-8535, Japan

**Keywords:** case report, Fogarty catheter, general anesthesia, supraglottic airway device, tracheoesophageal fistula

## Abstract

Surgery for tracheoesophageal fistula (TEF) often necessitates the insertion of a Fogarty catheter (FC) to assist the surgeon in identifying the fistula. However, when the TEF is located close to the glottis, bronchoscopic identification of the TEF and FC insertion can be particularly challenging. A 2-month-old female infant exhibited frequent apneic episodes during feeding from postnatal day 5. A subsequent contrast swallow study and bronchoscopy led to the diagnosis of H-type esophageal atresia. Surgical ligation of the TEF was performed on Day 64 of life. Under general anesthesia induction with endotracheal intubation, bronchoscopic visualization of the TEF was attempted before commencing surgery. However, the subglottic location of the TEF made its identification difficult. The TEF was subsequently successfully identified using a supraglottic airway device in combination with positive pressure ventilation. Thereafter, an FC was inserted through the supraglottic device, which was later replaced by tracheal intubation. No adverse events were observed during the procedure. The patient was extubated on postoperative day (POD) 6 and was discharged on POD 39. The method described in this report was effective for efficient insertion of the FC into the subglottic TEF and might contribute to safer and more reliable surgical performance.

## 1. Introduction

H-type esophageal atresia (EA) is a congenital variant of EA, accounting for approximately 4% of all cases. While the most common type, C-type EA, involves a blind-ending proximal esophagus with a tracheoesophageal fistula (TEF) between the trachea and distal esophagus, the H-type is characterized by the presence of a TEF without esophageal discontinuity, meaning both the trachea and esophagus remain patent [1, 2].

The TEF in C-type EA is often located within 2 cm of the carina, whereas in H-type EA, it tends to be located at the cervical level, i.e., near the upper trachea [2]. The high location of the TEF can make its identification challenging, even with the use of bronchoscopy via the endotracheal tube (ETT).

We describe a case in which we successfully identified a high TEF situated immediately below the glottis using a supraglottic airway (SGA) combined with the insertion of a Fogarty catheter (FC, Edwards Lifesciences, Irvine, CA, USA). This approach facilitated intraoperative TEF identification and ensured safe respiratory management without complications. Informed consent was obtained from the patient's parents for publication of this case report.

## 2. Case Presentation

The patient was a 2-month-old female, 54.9 cm in height, weighing 4.29 kg. She was born at 37 weeks and 2 days gestation, with a birth weight of 2997 g and an uneventful perinatal period. From postnatal day 5, she exhibited frequent apneic episodes during feeding. A contrast swallow study on Day 44 revealed a TEF at the level of Th1. Bronchoscopy performed via an SGA under general anesthesia on Day 48 identified a subglottic TEF after its dilation under positive pressure ventilation, confirming the diagnosis of H-type EA. The patient had no family history relevant to H-type EA.

On Day 63, TEF ligation via the cervical approach was planned under general anesthesia. Physical examination at this time revealed no significant findings. Preoperative blood tests, chest X-ray, and cardiac ultrasound were unremarkable. The surgeon requested the preoperative insertion of a catheter device for intraoperative TEF identification. Anesthesia was induced with 8-mg propofol, 8-μg fentanyl, and 4-mg rocuronium and maintained with total intravenous anesthesia using propofol (120–200 μg/kg/min) and remifentanil (0.5 μg/kg/min).

**Figure 1:**
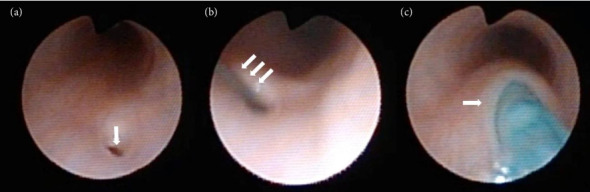
Findings of tracheal examination using bronchoscopy. (a) Orifice of the tracheoesophageal fistula (arrow). (b) Attempt to insert a Radifocus Guide Wire M (arrows) into the tracheoesophageal fistula. (c) Insertion of the Fogarty catheter into the tracheoesophageal fistula.

Prior to airway management, a 3 Fr FC was first placed through the glottis into the trachea using direct laryngoscopy (Miller blade #1.5) to aid with intraoperative identification of the TEF, followed by intubation with a 3.5-mm uncuffed ETT. A flexible bronchoscope (LF-P, Olympus, Japan) was inserted through the ETT to examine the upper tracheal lumen while gradually withdrawing the ETT in order to identify the TEF opening. However, because the TEF was located immediately below the glottis, the ETT was unintentionally withdrawn above the glottic level before the opening could be identified, resulting in accidental extubation. The TEF orifice, approximately 0.7 mm in diameter, was identified on the posterior wall of the trachea at the 5 o'clock position only during manual positive pressure ventilation after airway reestablishment using an i-gel SGA (size 1, Intersurgical, UK) (Figure 1(a)).

**Figure 2:**
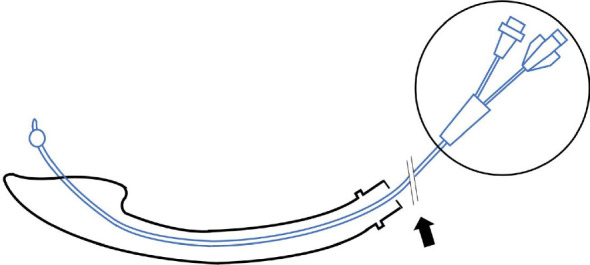
To facilitate removal of the supraglottic airway device, the external inflation port of the Fogarty catheter was cut (black arrow), and the port was removed (black circle).

**Figure 3:**
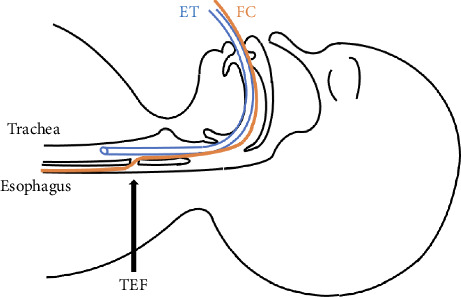
Schematic diagram showing the Fogarty catheter passing through the tracheoesophageal fistula. ET: endotracheal tube, FC: Fogarty catheter, TEF: tracheoesophageal fistula.

Attempts to insert a 0.018-inch (0.46 mm) Radifocus guidewire (Terumo, Japan) into the TEF via the SGA were unsuccessful due to the flexibility of the wire and the small size of the TEF orifice (Figure 1(b)). Instead, an FC was inserted through the SGA into the TEF (Figure 1(c)), and its position within the stomach was confirmed under fluoroscopy. After the FC was fixed in place, it was found that external inflation port of the FC was too large to pass through the SGA lumen, so this port of the FC was cut to facilitate SGA removal (Figure 2). Subsequently, oral intubation was performed under direct laryngoscopy (Figure 3). The entire procedure took 36 min and was performed without complications, such as ventilation difficulty, hypoxemia, or hypercapnia. TEF ligation surgery was completed in 2 h and 36 min without adverse intraoperative events. The patient was extubated on postoperative day (POD) 6 after being transferred to the neonatal intensive care unit with transient vocal cord paralysis that improved over time and was discharged on POD 39.

## 3. Discussion

We managed anesthesia for a subglottic TEF using the combination of an SGA for FC insertion and positive pressure ventilation via an ETT, without complications such as hypoxemia.

The use of an SGA for TEF identification near the glottis proved valuable. Traditionally, TEF confirmation in EA is often performed under bronchoscopy through an ETT [1]. However, when the TEF is occluded by the ETT, its identification can be challenging. Additionally, positive pressure ventilation through the ETT risks insufflation of the stomach. In our case, the small subglottic TEF was difficult to identify, and device insertion via the ETT was also challenging. Therefore, we planned to first attempt TEF identification through the ETT, with the SGA as a backup. Although TEF identification via the ETT was not successful, switching to an SGA allowed for continuous ventilation with minimal interruptions. Positive pressure ventilation through the SGA facilitated TEF dilation, making it easier to identify, which was a major advantage.

Regarding the choice of devices for TEF insertion, the FC proved more useful than the guidewire. In this case, we initially selected the guidewire due to the small size of the TEF, but the flexible guidewire failed to pass through the orifice, probably because the 0.018-inch guidewire was too soft. The FC was easier to guide into the posteriorly located TEF due to its curved tip and appropriate firmness. Although the difference in insertion success was likely due to the characteristics of the device materials, it is possible that a thicker guidewire would have been more successful.

There are various methods for airway management, ventilation, and TEF device insertion in EA, which are influenced by the location and size of the TEF. Zhou et al. reported a technique in a 1-day-old infant with EA with TEF, where they first inserted a bronchial blocker into the trachea under direct laryngoscopy, followed by securing the airway with an SGA. They then guided the bronchial blocker into the TEF under visualization by a bronchoscope passed through the SGA [3]. We did not choose this technique due to concerns about the small TEF and potential for bronchial blocker interference with SGA positioning, which could hinder ventilation. We instead used an FC, which was beneficial in maintaining airway control and adjusting positive pressure ventilation, as needed. Thus, device selection should be tailored to each case, considering both the maneuverability of the device and its impact on ventilation.

This report highlights the ease of subglottic TEF identification through FC insertion under positive pressure ventilation via the SGA, which would have been difficult with an ETT. Gastric insufflation during positive pressure ventilation is of significant concern during the anesthesia management of EA with TEF. In C-type EA, this can lead to gastric distension, diaphragmatic elevation, poor ventilation, or even gastric rupture. This makes it essential to avoid high positive pressures based on the preoperative respiratory status, TEF location, and size. In such cases, spontaneous breathing, low-pressure ventilation, or FC insertion into the TEF should be considered. Techniques for preserving spontaneous breathing in pediatric patients include the use of propofol in combination with inhalational anesthetics or ketamine [4, 5].

On the other hand, positive pressure ventilation can be relatively safe even in cases of EA, when the TEF is small [6]. In this case, FC insertion into the TEF likely prevented gastric insufflation, allowing for stable positive pressure ventilation. However, since we inserted the FC via an SGA, the FC cuff was not used due to the need for removing the hub for SGA removal. In this case of H-type EA, the esophagus was patent, allowing gastric decompression via a nasogastric tube so that gastric insufflation was not a concern. However, in cases with larger TEFs, gastric insufflation might be uncontrollable, and this method might not always be appropriate.

The purpose of FC insertion in this case was to assist with intraoperative TEF identification. Subjectively, the surgeon found this technique useful particularly when managing narrow or difficult-to-localize TEFs. By passing an FC through the fistula, intraluminal support is provided, which improves tract delineation and facilitates intraoperative identification and repair. However, objective evaluation of its utility is challenging. TEF device placement during surgery has been reported to aid in achieving repair via the cervical approach without thoracotomy, suggesting that device placement might have contributed to the smooth progression and success of the surgery and the reduced surgical time in this case [7, 8]. However, as this is a case report, improvements in objective metrics such as reduced surgical time cannot be definitively attributed to this technique without comparison to a control series. In addition, TEF device insertion should not be insisted upon if it compromises respiratory management.

In conclusion, the method described in this report might prove effective for the safe and efficient insertion of an FC into a subglottic TEF.

## Data Availability

The data that support the findings of this study are available from the corresponding author upon reasonable request.
